# Thermal conductivity and air-mediated losses in periodic porous silicon membranes at high temperatures

**DOI:** 10.1038/s41467-017-00115-4

**Published:** 2017-09-04

**Authors:** B. Graczykowski, A. El Sachat, J. S. Reparaz, M. Sledzinska, M. R. Wagner, E. Chavez-Angel, Y. Wu, S. Volz, Y. Wu, F. Alzina, C. M. Sotomayor Torres

**Affiliations:** 1 Catalan Institute of Nanoscience and Nanotechnology (ICN2), CSIC and BIST, Campus UAB, Bellaterra, 08193 Barcelona, Spain; 2 NanoBioMedical Centre, Adam Mickiewicz University, ul. Umultowska 85, PL-61614 Poznan, Poland; 3 Max Planck Institute for Polymer Research, Ackermannweg 10, 55218 Mainz, Germany; 4 Department of Physics, Universitat Autonoma de Barcelona, Campus UAB, Bellaterra, 08193 Barcelona, Spain; 5 Laboratoire dEnergetique Moleculaire et Macroscopique, Combustion, CNRS, CentraleSupelec, Grande Voie des Vignes, 92295 Chatenay-Malabry, France; 6 Laboratory for Integrated Micro-Mechatronics Systems, CNRS UMI2820, Institute of Industrial Science, University of Tokyo, 4-6-1 Komaba, Meguro-ku, Tokyo, 153-8505 Japan; 7 ICREA Pg. Lluís Companys 23, 08010 Barcelona, Spain; 8 Institut de Ciència de Materials de Barcelona, ICMAB-CSIC, Campus Universitari de Bellaterra, E-08193 Bellaterra, Spain; 9 Institute of Solid State Physics, Technische Universität Berlin, Hardenbergstr. 36 10623 Berlin, Germany

## Abstract

Heat conduction in silicon can be effectively engineered by means of sub-micrometre porous thin free-standing membranes. Tunable thermal properties make these structures good candidates for integrated heat management units such as waste heat recovery, rectification or efficient heat dissipation. However, possible applications require detailed thermal characterisation at high temperatures which, up to now, has been an experimental challenge. In this work we use the contactless two-laser Raman thermometry to study heat dissipation in periodic porous membranes at high temperatures via lattice conduction and air-mediated losses. We find the reduction of the thermal conductivity and its temperature dependence closely correlated with the structure feature size. On the basis of two-phonon Raman spectra, we attribute this behaviour to diffuse (incoherent) phonon-boundary scattering. Furthermore, we investigate and quantify the heat dissipation via natural air-mediated cooling, which can be tuned by engineering the porosity.

## Abstract (web summary)

Nanostructuring of silicon allows acoustic phonon engineering, but the mechanism of related thermal transport in these structures is not fully understood. Here, the authors study the heat dissipation in silicon membranes with periodic nanoholes and show the importance of incoherent scattering.

## Introduction

Progress in the last few decades in nano-scale thermal transport has enabled a significant degree of control over heat and sound propagation by lattice vibrations—phonons. The latest investigations on the thermal properties of silicon, the most common material in electronics, micro-electro-mechanical system and nano-electro-mechanical systems (NEMS) and photonics, have pointed to nanostructuring as a highly efficient approach to acoustic phonon engineering^1–7^. In this context, the reduction of the characteristic sizes in nanowires and ultra-thin membranes, surface nano-engineering and patterning of holes in thin membranes have been used to modify the acoustic phonon dispersion and lifetimes of relevance for hypersound and heat transport^2, 5, 7^. On the one hand, structures with periodically arranged holes have shown coherent features, such as a modified phonon dispersion relation with band folding, and therefore they can be termed as truly phononic crystals (PnCs). On the other hand, single crystalline porous silicon membranes, with sub-micrometre feature sizes, suppressed lattice thermal conductivity *κ* and potentially encouraging power factor, are considered as a practical realisation of the phonon-glass electron-crystal concept and therefore are expected to enhance the thermoelectric performance or the figure of merit (ZT) of silicon^8^. Moreover, they offer the possibility of modifying the temperature dependence of *κ* by nanostructuring and thereby provide a tool to control the directional heat flow to enable, e.g., heat rectification schemes^9–11^. Nevertheless, the phenomenon of the reduced thermal conductivity in periodic porous membranes (or PnCs) and its physical origin, i.e., the contribution of coherent (wave-like) and incoherent (particle-like) processes, the role of structure porosity, lattice parameter, disorder and surface roughness are still a matter of a lively debate based on a variety of theoretical models and experimental techniques. Up to now all the experimental efforts, made mostly by using suspended heater-thermometer platforms^8, 12–15^ or, more recently, time domain thermoreflectance^16, 17^, have focused on a temperature range close or below room temperature (RT). For example, the results obtained so far clearly show that the reduction of *κ* is correlated with the material porosity, which cannot be explained by considering simply volume removal. However, both techniques are burdened with assumptions regarding interface thermal resistance and boundary conditions that require additional modelling and free-fitting parameters. Furthermore, they are not suitable for the high temperature investigation of the thermal properties, crucial for most applications with a heat sink set at RT, and the possible contribution of air to heat dissipation. In the regime where the surface-to-volume ratio is high, the latter may dominate heat flow resulting in thermal losses. Therefore, the balance between the influence of air on the efficiency of the thermoelectric modules and the cost of using evacuated modules have to be considered. On the other hand, passive and efficient air-mediated cooling could be considered as an advantage for heat dissipation in nanodevices. The miniaturisation and resulting size-induced reduction of the thermal conductivity may have adverse consequences to remove fast and efficiently the redundant heat caused by Joule or radiative heating. This may lead to overheating of the functional units in silicon-based electronics, NEMS, photonic and optomechanical devices, and thereby result in, e.g., a decrease of computational performance, signal processing efficiency and the quality factor or in a drift of the operational frequency.

In this work we report a new experimental insight into the in-plane thermal properties of periodic porous silicon membranes, with a special focus on the reduction of the thermal conductivity and its evolution at high temperatures. Furthermore, we investigate natural air-mediated cooling and demonstrate its impact on heat dissipation in the considered structures. To overcome disadvantages coming from techniques requiring electrical contacts, transducers or data analysis with free-fitting parameters, we employ the contactless technique of two-laser Raman thermometry (2LRT)^18, 19^.

## Results

### Experimental techniques

2LRT has recently been shown to be a suitable method for thermal mapping with sub-micrometre spatial resolution and for determining *κ* of ultra-thin silicon membranes^18^, including the effect of the native oxide^19^. In principle, it can be applied to any material in membrane format, where the absorbed power can be considered uniform along the thickness, exhibiting a detectable temperature-dependent phonon Raman scattering signal.

**Fig. 1:**
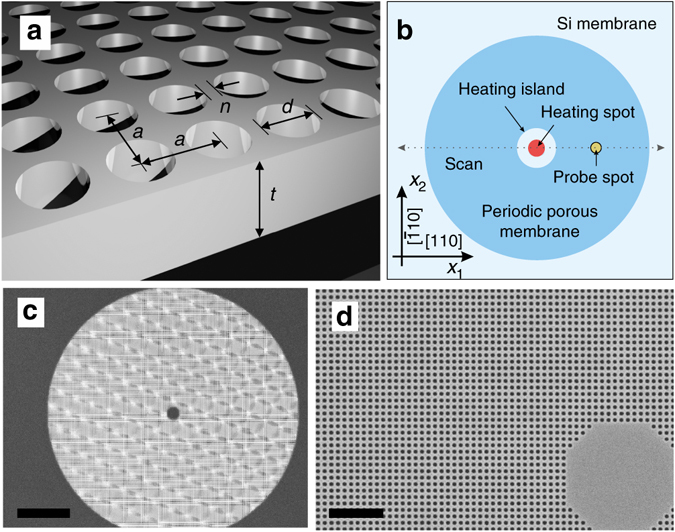
Samples for two-laser Raman thermometry experiment. **a** Schematic picture of the periodic porous membrane—square lattice of cylindrical holes in the free-standing membrane, where *t* = 250 nm is the membrane thickness, *d* is the hole diameter, *a* is the lattice parameter and *n* stands for the neck. **b** Top view of the sample design depicting the temperature scan direction and crystallographic orientation. The porous area is enclosed by two circles with diameters of about 5 and 100 μm. **c**, **d** Scanning electron microscope images of sample S2 (*a* = 250 nm and *d* = 140 nm). *Scale bars* in **c**, **d** are 20 and 2 μm, respectively

The fabrication of periodic porous membranes made of a square lattice of cylindrical holes (Fig. 1) in the membrane was based on electron beam lithography and reactive ion etching. As the basis and reference sample we used commercially available, single crystalline silicon (001), *t* = 250 nm thick membranes with a window size of 3.2 × 3.2 mm^2^ placed on a thick silicon square frame. Three samples labelled S1, S2 and S3 of an intentionally similar hole diameter of about *d* = 135 nm differ in the lattice constant *a* measured as 300, 250 and 200 nm, respectively. Figure 1b displays a schematic top view of the sample designed to keep radial symmetry about the *x*_3_ axis running through the heating spot. The heating island of a diameter about 5 μm has no holes to avoid the diffraction of the heating beam and subsequent uncertainties in the absorbed power measurements. This island is surrounded by the periodic porous membrane with a total diameter of 100 μm. In general, the radial symmetry of the sample simplifies measurements to a single line scan in the *x*_1_ direction, which determines a temperature profile. Figure 1c and d shows typical scanning electron microscope (SEM) images of the samples with a visible heating island. For details see Methods Section.

### Thermal conductivity of pristine membrane

**Fig. 2:**
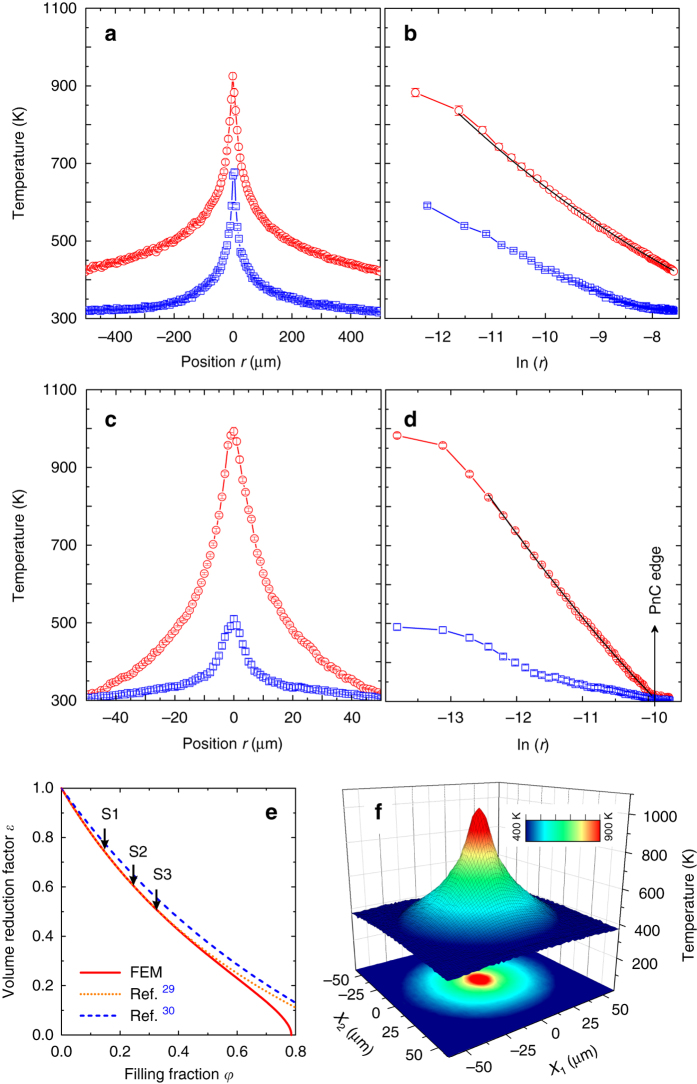
Two-laser Raman thermometry results. Linear and corresponding logarithmic temperature profiles of **a**, **b** pristine 250 nm thick silicon membrane and **c**, **d** sample S3 with lattice parameter of *a* = 200 nm and hole diameter of *d* = 130 nm. *Red circle-line* and *blue square-line plots* indicate experimental data obtained in vacuum and air, respectively. *Solid lines* in **b**, **d** denote theoretical fits using Eq. (2). *Error bars* in **a**, **b**, **c**, **d** represent experimental uncertainties of the measured temperature (see Methods Section). **e** Volume reduction factor *ε* as a function of porosity calculated by FEM and using analytical expressions. **f** Measured temperature map of the sample S1 with lattice parameter *a* = 300 nm and hole diameter *d* = 135 nm

Although the reduction of the thermal conductivity in silicon membranes or thin films is well studied, there is a lack of experimental data at temperatures above 400 K^20^. Moreover, the reported values of *κ* at RT vary over a wide range, e.g., for 250 nm thick membranes the values reported go from 60 to 100 W m^−1^ K^−1^ ^20–25^. Thus, we first characterise the bare membrane pointing out at the same time the principles of the experimental approach. Figure 2a displays two steady-state heat flow temperature profiles obtained at the same heating power *P*_0_ = 8.125 mW, but under different ambient conditions. The plots labelled vacuum and air correspond to the ambient pressure of 10^−3^ and 10^3^ mbar, respectively. These measurements were also performed in the porous samples to ascertain the effect of the air-mediated cooling discussed later. It follows from Fig. 2a that by applying a relatively small power to the membrane in vacuum a temperature difference from 920 to 420 K is established over a distance of 500 μm which, in practice, allows us to determine *κ* as a function of temperature over a wide range by a single measurement. To do so we assume the temperature gradient to be zero in the *x*_3_ direction and diffusive in-plane heat flow, for which the radial heat is governed by Fourier’s law: *P*_0_/(2*πrt*) = −*κ*(*T*)d*T*/d*r*, where *r* is the distance from the centre and *t* is the membrane thickness. Then, taking $r{\rm{d}}T/{\rm{d}}r = {\rm{d}}T/{\rm{d}}(\ln r) = \xi (r)$ we obtain the following expression for the thermal conductivity as a function of temperature:

1 $$\kappa \left( T \right) = - \frac{{{P_0}}}{{2\pi t{\it{\xi }}\left( r \right)}}.$$

**Fig. 3:**
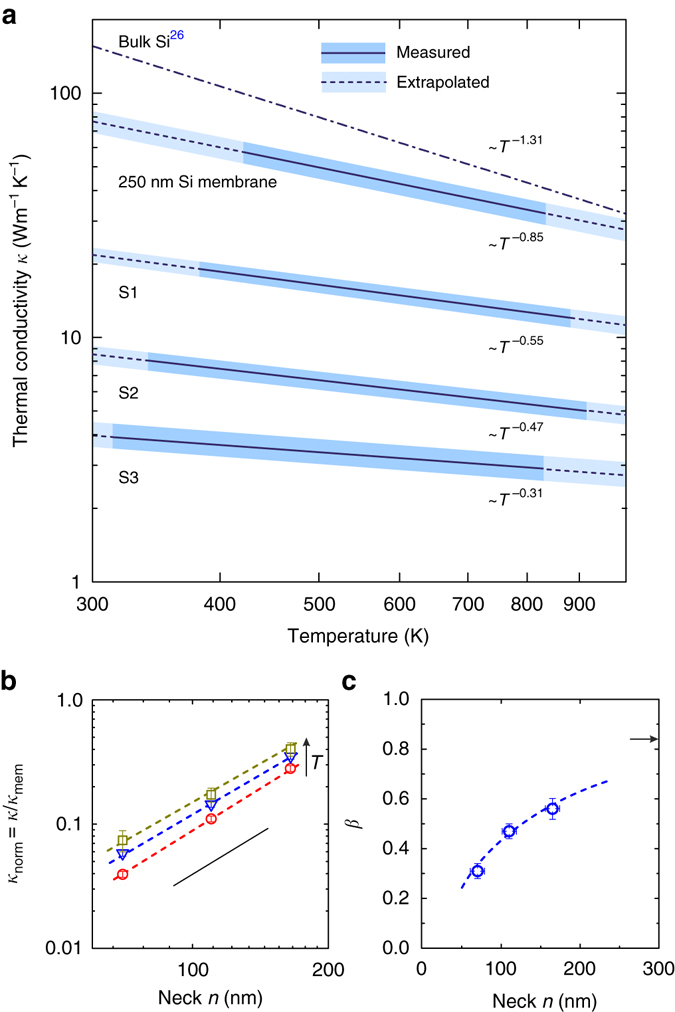
Temperature dependence of the thermal conductivity. **a** Thermal conductivity of porous membranes (S1, S2 and S3) and 250 nm thick membrane as a function of temperature. *Solid* and *dashed lines* denote measured and extrapolated *κ*, respectively. The shaded areas indicate experimental uncertainty derived in Methods Section. **b** Normalised thermal conductivity of porous membranes as a function of the neck size *n* for three example temperatures; *circles*—300 K, *triangles*—600 K, *squares*—900 K. The *error bars* are experimental uncertainties of *κ* derived in Methods Section. The *dashed lines* are guides to the eye connecting data points of the same temperature. The *solid line* is a reference plot indicating *n* ^2^ dependence. **c** The exponent *β* governing the temperature dependence of *κ* as a function of the neck size *n*. The *dashed line* is a guide to the eye and the arrow indicates *β* of the 250 nm thick membrane. *Horizontal* and *vertical error bars* indicate the experimental uncertainties of *β* and *n* determined from the data fit using Eq. (2) and from scanning electron microscope images, respectively

Consequently, knowing *P*_0_ and *t*, the value of *κ*(*T*) can be extracted from the logarithmic temperature profile *T*(ln *r*) shown in Fig. 2b. For the simplest case where *κ* ≠ *f*(*T*), *κ* would be directly obtained from Eq. (1) and the slope of the linear fit of the logarithmic temperature profile. Nevertheless, *T*(ln *r*) plotted in Fig. 2b is clearly nonlinear and, for convenience, we use a specific function to fit the experimental data. Assuming that *κ*(*T*) of the membrane and the porous samples resembles the behaviour of bulk silicon at high temperatures, we put *κ*(*T*) = *αT* ^−*β*^ in Fourier’s law^26^, which after integrating, gives the function:

2 $$T = {\left( {A - \frac{{{P_0}\left( {1 - \beta } \right)}}{{2\pi t\alpha }}\ln \,r} \right)^{1/\left( {1 - \beta } \right)}},$$

where *A* is a constant of integration while the fitting parameters *β* and *α* determine *κ*(*T*). The curve in Fig. 2b is obtained by fitting Eq. (2) to the experimental data points. We note that this procedure is valid for a data range starting at a distance of a few micrometres from the central point *r* = 0. This is due to the Gaussian shape of the heating source and possible effects of the photo-generated free charge carriers at high concentration, which may diffuse away from the illuminated area and contribute to the total thermal conductivity with two counteracting effects^27^. The data points that do not match a preliminary fit using Eq. (2), and therefore from the sample area where the assumed dependence of *κ*(*T*) does not apply are thus omitted. In this study we use the data obtained for *r* > 3 μm, for which we assume the role of the lattice thermal conductivity is predominant and we impose its power function dependence. The effect of thermally and photo-generated electron–hole pairs is discussed in Methods Section. The determined *κ*(*T*) is plotted in Fig. 3a and assuming as before *κ*(*T*) = *αT* ^−*β*^, extrapolated to close the ends of the 300−1000 K range. An unambiguous reduction of the thermal conductivity and of its dependence on temperature with respect to bulk silicon^26^ is clearly observed. This behaviour is commonly attributed to the phonon mean free path (MFP) suppression due to the phonon diffuse boundary scattering in addition to phonon–phonon scattering processes^7, 19, 21, 22, 24, 25^. From Fig. 3a we obtain *κ*_mem_ = 78 ± 6 W m^−1^ K^−1^ for the pristine membrane at 300 K, which corresponds to a two-fold reduction of *κ* with respect to bulk Si. This value is in good agreement with recent transient thermal grating measurements, on silicon membranes^25^. Furthermore, it is consistent with other existing data obtained for silicon thin films of similar thicknesses by means of the harmonic Joule heating technique^23^. A further decrease of *κ* can be achieved by reducing the membrane thickness and by surface nano-engineering down to a value of 8 ± 2 W m^−1^ K^−1^ for 9 nm thick membrane, as recently reported^19, 24^.

### Thermal conductivity of periodic porous membranes

**Fig. 4:**
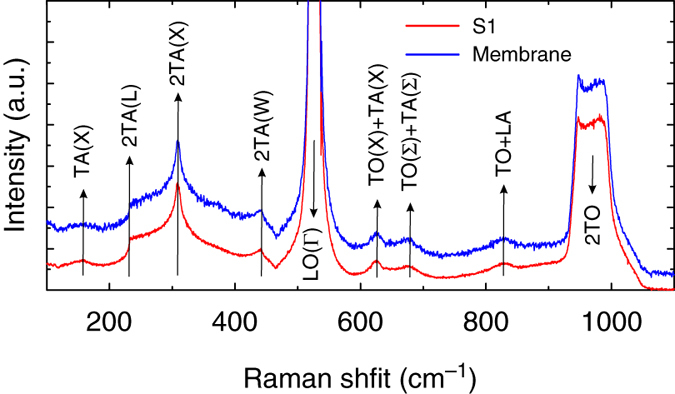
One- and two-phonon Raman spectra. Data obtained for pristine 250 nm membrane and S1 at room temperature in ${x_3}\left( {{x_1}{x_1}} \right){\bar x_3}$ scattering geometry. The *arrows* indicate critical points of the first Brillouin zone of bulk silicon, where TA and TO are transverse acoustic and optical modes, respectively, and LO are longitudinal optical modes^38^

Now we shall move on to the results obtained in porous membranes in vacuum. As previously formulated for the case of the bare membrane, we use the temperature line scan and the corresponding temperature profile in logarithmic scale to determine *κ*(*T*). These are shown in Fig. 2c and d, respectively, for the case of the sample S3 (Table 1). For the in-vacuum measurements we notice that by heating with less power than for the pristine membrane, namely *P*_0_ = 0.41 mW, we create a similar temperature rise at *r* = 0. Simultaneously, the created temperature gradient covers a range of about 300–1000 K over a distance of only 50 μm. This suggests a reduction of *κ* caused by the lattice of holes in the membrane. In principle, this effect could result from volume removal, while the intrinsic material properties such as the thermal conductivity might be preserved. Therefore, *κ*_exp_(*T*) of the porous membranes determined from the experimental data and Eq. (2) has to be scaled by a factor, which takes into account the specific porosity of the membrane. This can be done by using a volume correction factor *ε* that can be obtained analytically or, more accurately, numerically by solving the diffusive heat transport model using the finite element method (FEM)^28^. Figure 2e shows a plot of *ε* as a function of the sample filling fraction *ϕ* obtained from FEM, which we use here, compared with two analytical expressions by Eucken^29^ and later by Hashin and Shtrikman^30^. Once *κ*_exp_(*T*) and *ϕ* of the porous membrane are obtained, we calculate the intrinsic thermal conductivity *κ*(*T*) from the formula: *κ*(*T*) = *κ*_exp_(*T*)/*ε*(*ϕ*). It is worth mentioning that Eq. (2) used to determine *κ*_exp_(*T*) is valid when the radial symmetry of the temperature field is preserved. That symmetry is preserved for the pristine silicon membranes as reported in ref. ^18^. In such a case the in-plane thermal conductivity of the membrane follows *κ* of bulk silicon in diffusive regime that is isotropic. This is not obvious for the periodic porous membranes with the in-plane structural anisotropy driven by the square lattice of holes. Nevertheless, in our case, the 2D thermal map obtained for the sample S1 and shown in Fig. 2f clearly demonstrates radial symmetry with no signature of the structure driven in-plane thermal anisotropy, and thus validates the use of Eq. (2). We note that the probe laser spot measures an average temperature over its size, which comprises several unit cells. In this way we obtain an effective *κ* of porous membranes, which is the thermal conductivity that would correspond to a homogeneous membrane in the Fourier approximation. Figure 3a shows a comprehensive plot of *κ*(*T*) obtained for three porous samples and the 250 nm thick bare membrane. These results show the reduction of the thermal conductivity in the porous samples with respect to the pristine membrane, which depends on the sample feature size and temperature. It is commonly accepted that the observed reduction of the thermal conductivity in the periodic porous membranes is dominated by the diffuse phonon-boundary scattering, but the contribution of coherent effects seems to remain a matter of ongoing debate^2, 5, 13, 15, 28, 31^. It is clear that phonons with MFP shorter than the lattice parameter can be treated purely as particles governed by the bulk dispersion relation. Otherwise, the wave-like nature can be manifested as, e.g., dispersion relation zone folding, flattening of branches and band gaps, all of which have implications on the phonon group velocity and density of states (DOS) and consequently on the thermal conductivity. In such a case periodic porous membranes are synonymous to 2D PnCs. In bulk silicon, MFPs span a broad range with MFPs longer than 1 μm contributing to ~ 50% of the total thermal conductivity^25, 32–34^. Furthermore, as predicted theoretically, in the case of 250 nm thick silicon films, phonon MFPs exceeding 300 nm contribute to about 20% of the total thermal conductivity^35^. In this work we investigate structures with lattice parameters smaller than 300 nm. Therefore, it might be reasonable to consider the suppression of the thermal conductivity due to coherent Bragg scattering. Indeed, the results of Brillouin light scattering (BLS) from similar structures showed unambiguously the modification of the phonon dispersion, exhibiting typical features of PnCs band diagram, such as zone folding, modification of group velocity and band gap opening^36^. However, BLS probes the coherence in a relatively low frequency range (up to tens of GHz), which becomes relevant at very low temperatures, where most of the heat is carried by long wavelength phonons^5, 17, 37^. At high temperatures their contribution is negligible and, following recent theoretical work, most of the heat in bulk silicon is carried by phonons of frequencies around 5 THz^31, 32^. Although this range is beyond BLS, the change in the phonon dispersion relation in periodic porous membranes (or PnCs), if any, can be captured indirectly by two-phonon (second order) Raman scattering^38–42^. The second-order Raman spectrum, which in bulk silicon is dominated by overtones and, thus, has a striking similarity with the two-phonon DOS, is sensitive to the phonon dispersion relation along the Brillouin zone. Although it cannot be used to determine the dispersion relation, any change (additional dips or peaks) may be a clear indication of the modified phonon dispersion. In Fig. 4 we compare second-order Raman spectra of the pristine membrane and S1 measured in ${x_3}\left( {{x_1}{x_1}} \right){\bar x_3}$ scattering geometry. As we can notice the measured range of 100–1100 cm^−1^ that corresponds to 1.5–16.5 THz shows no detectable singularities in the Raman spectra of the S1 compared to the pristine membrane. Furthermore, all spectral features are typical of bulk Si. Thus, it is unlikely that coherent effects play a role in the observed reduction of *κ*. We note that the latest results obtained at RT provide contradictory conclusions and the comprehensive understanding of this problem is still elusive^15, 43^. As follows from Fig. 3a, at first glance *κ*(*T*) decreases together with the sample neck size, namely, the shortest distance separating holes, given by *n* = *a*−*d*. Figure 3b depicts explicitly this behaviour in a log–log plot of the normalised thermal conductivity *κ*_norm_ at three example temperatures as a function of the neck size. From these data we can conclude that over a broad range of temperatures the dependence of *κ*_norm_ on the neck size can be approximated by a function *k*_norm_ ∝ *n* ^2^. Interestingly, the same correlation function has been found recently for similar structures, albeit smaller feature size^8^. This trend holds for three example temperatures and for a particular neck size the rate of decrease of *κ* becomes smaller with increasing temperature. Since in our case the thermal conductivity depends on temperature as *κ*(*T*) ∝ *T* ^−*β*^ we can conclude that this behaviour is governed by the exponent *β*. The latter parameter is plotted as a function of neck size in Fig. 3c. As can be seen, the temperature dependence of *κ* becomes weaker with decreasing distance between holes. This points to the diminishing role of phonon–phonon processes, which are overwhelmed by the temperature-independent diffuse phonon-boundary scattering. From Fig. 3b and c we deduce that both, the reduction and temperature dependence of the thermal conductivity, are related to the geometrical features of the samples. In other words, by changing the neck size, the thermal properties can be designed and tuned in a simple and efficient manner. All the results discussed above are summarised in Table 1. We point out that the thermal conductivity of the porous membranes can be reduced at most by a factor of about 40 with respect to bulk silicon at 300 K, thus approaching the amorphous limit of silicon *κ*_a-Si_ = 1.7 W m^−1^ K^−1^ ^34^.

**Table 1:** Characteristic sizes, coefficients and example experimental data for the porous membranes and the 250 nm silicon membrane: *a*—lattice spacing, *d*—hole diameter, *ϕ*—filing fraction, *ε*—volume reduction factor, *κ*—thermal conductivity (W m^−1^ K^−1^).

Sample	*a* (nm)	*d* (nm)	*ϕ*	*ε*	*κ* at 300 K	*κ* at 600 K	*κ* at 900 K	*β*	*κ*_bulk_/*κ* at 300 K
Membrane	–	0	0	1	77.9 ± 8.1	42.5 ± 4.3	30.1 ± 3.2	0.85	~2
S1	300	135	0.159	0.725	21.9 ± 1.9	14.6 ± 1.1	11.9 ± 0.9	0.55	~7
S2	250	140	0.246	0.604	8.5 ± 0.9	6.2 ± 0.6	5.1 ± 0.5	0.47	~18
S3	200	130	0.332	0.499	3.9 ± 0.4	3.2 ± 0.3	2.8 ± 0.3	0.31	~40

Experimental uncertainties of *κ* are calculated using the error propagation described in Methods Section.

### Air-mediated cooling

Up to this point, we have considered only measurements performed in vacuum, i.e., where air-mediated thermal transport via convection and conduction is negligible. Air-mediated heat transport becomes rather important for the porous membranes as operational building blocks in real-life applications, besides those for outer space. In principle, air has a poor thermal conductivity of about 2.623−6.763 × 10^−2^ W m^−1^ K^−1^ between 300 and 900 K^44^. However, in structures with high surface-to-volume ratio and reduced thermal conductivity heat dissipation via air has to be taken into account. This effect was previously observed in graphene^45^, photonic crystals^46^ and nano beams^47^. To analyse this issue in porous membranes we go back to Fig. 2a–d, where we compare 2LRT results obtained in vacuum and air. At first glance, the temperature profiles of the pristine membrane and the example porous membrane shown in Fig. 2a and c, respectively, clearly indicate heat losses caused by the presence of air. Foremost, the temperature rise at *r* = 0 for both samples in air are significantly smaller than those measured in vacuum. Consequently, if one were to use the conventional single laser Raman thermometry the thermal conductivity measured in air would be overestimated by factors of about 1.4 and 1.9 for the membrane and S3, respectively. Likewise, *κ*(*T*) obtained from the corresponding logarithmic temperature profiles recorded in air (Fig. 2b and d) would be inaccurate.

In what follows, we use these data and propose an approach to quantify the heat dissipation resulting from natural air-mediated cooling in 2D systems. Let us return to Eq. (1), which determines *κ* in vacuum conditions at a temperature *T* using the measured *P*_0_ and *ξ*(*r*). The latter, considered simply as the slope of the logarithmic temperature profile at *r*, is different for the vacuum and air data, showing larger nonlinearity for data taken in air, as seen in Fig. 2b and d. The only reason for this difference comes from the air-mediated losses, denoted *P*_loss_, which occur in a distance from 0 to *r*. The thermal conductivity *κ*(*T*) and thickness *t* remain unchanged, thus in the case of the data obtained in air Eq. (1) can be rewritten as:

3 $$\kappa \left( {T'} \right) = - \frac{{{P_0} - {P_{{\rm{loss}}}}}}{{2\pi t{\it{\xi '}}\left( r \right)}},$$

where we denote the temperature and the slope of the logarithmic temperature profile at any arbitrary point *r* determined in air by *T*′ and *ξ*′(*r*), respectively. By combining Eqs (1) and (3) we obtain a simple expression that determines the relative air-mediated losses:

4 $$\frac{{{P_{{\rm{loss}}}}}}{{{P_0}}} = 1 - \frac{{{\it{\xi '}}\left( r \right)}}{{{\it{\xi }}\left( r \right)}}\frac{{\kappa \left( {T'} \right)}}{{\kappa \left( T \right)}},$$

**Fig. 5:**
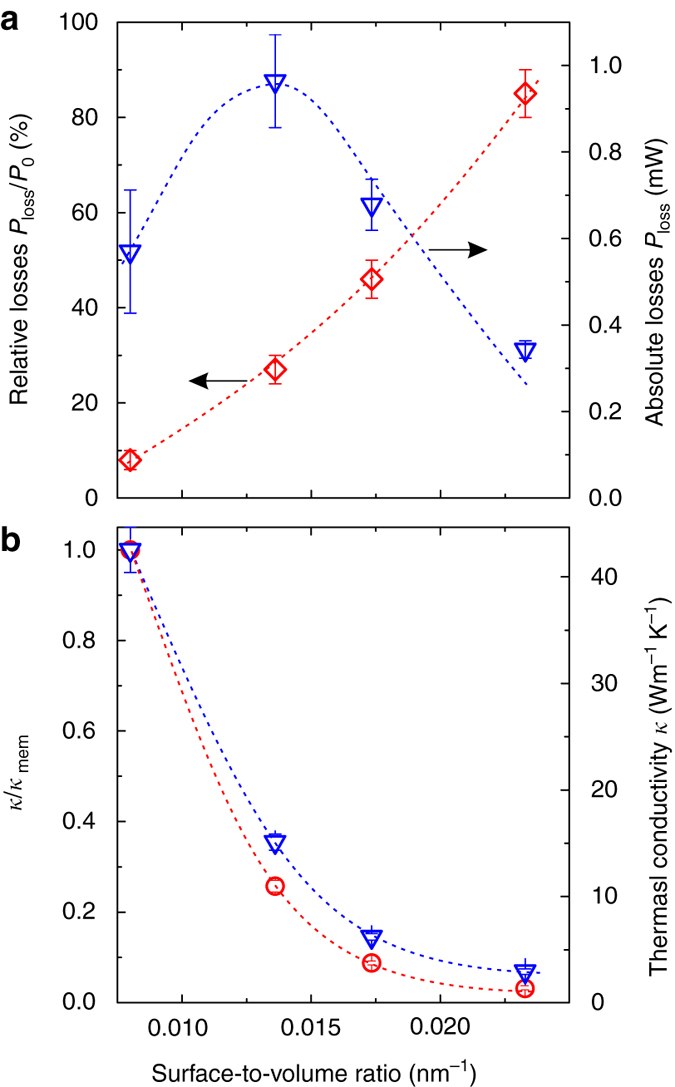
Air-mediated heat losses. **a** Relative (*red diamonds*, *left axis*) and absolute (*blue triangles*, *right axis*) losses caused by air-mediated cooling as a function of surface-to-volume ratio. **b** Measured *κ*_exp_ = *κε* (*red circles*) and intrinsic *κ* (*blue triangles*) thermal conductivity at 600 K as a function of surface-to-volume ratio. The *dashed lines* in **a**, **b** are guides to the eye. The *errors bars* are derived using the error propagation of *κ* derived in Methods section

Figure 5a depicts relative losses as a function of the surface-to-volume ratio calculated for the membrane and the three porous samples. Here, we compare heat dissipation from the lateral area defined by the porous membrane, thus Eq. (4) is derived for *r* = 50 μm. The losses are seen to vary from about 8 to 85%, while the general trend is clearly nonlinear. The latter may result from the porosity and specific orientation of the samples, in particular the holes, with respect to the gravity vector, which allows free air flow through the holes. Consequently, the losses grow faster than it would be expected from the increasing surface-to-volume ratio. In general, one can conclude from these data that air results in significant heat dissipation which, for the porous and thin membranes, cannot be neglected. Unfortunately, this limits the applicability of these structures in low-cost thermoelectric devices, which have to operate at atmospheric pressure with a reasonable performance. Nevertheless, this clear disadvantage might be favourable in cases where fast and efficient cooling is required like in silicon-based electronics, mechanical resonators, photonics and optomechanics. To analyse this issue we plot in Fig. 5a the absolute value of losses *P*_loss_ as a function of surface-to-volume ratio. In this case, the use of relative losses may result in a misleading conclusion, where the optimum surface-to-volume ratio would need to be as large as possible. As shown before the measured samples differ in the thermal conductivity, therefore, to get approximately the same temperature rise at the hot spot we applied a different heating power for each case. From Fig. 5a we find that the porosity of S1 increases the absolute losses by about 60% with respect to the pristine membrane, but this trend is reversed when the surface-to-volume ratio is further increased (samples S2 and S3). To understand this behaviour we examine the measured *κ*_exp_ and the intrinsic *κ* thermal conductivity, which are plotted at example temperature of 600 K in Fig. 5b. The extensive structure property such as the thermal conductance is governed not only by the intrinsic *κ* but also by the volume removal factor *ε*, thus the effective thermal conductivity values of the porous membranes are even lower and given by *κ*·*ε*. Now, if we compare Fig. 5a and b we see that increasing surface-to-volume ratio results in two opposite effects: the increase of the relative heat losses due to the presence of air and the reduction of the thermal conductivity. In other words, if efficient heat removal is the main priority then the effect of the reduced *κ* has to be minimised. In practice this boils down to making the porous membrane neck sufficiently larger than the thermal phonon MFP.

## Discussion

In summary, we investigated heat transport carried via lattice conduction and air convection and conduction in porous membranes made of square array of holes in 250 nm thick monocrystalline silicon membranes by 2LRT. We show that the in-plane thermal conductivity of silicon and its temperature evolution from RT to about 900 K can be effectively reduced and tuned by means of the sample geometrical feature, i.e., the neck size. We also demonstrate that the thermal conductivity at RT can be decreased down to about 4 W m^−1^ K^−1^. This value reaches the amorphous limit of silicon and to be achieved in a pristine monocrystalline silicon membrane it would require a thickness below 9 nm^19, 24^. The measured two-phonon Raman spectra of a pristine membrane and of periodic porous membranes show no significant difference, and therefore we attribute the observed reduction to the shortening of the phonon MFP due to diffuse (incoherent) phonon-boundary scattering. Furthermore, we determine the heat dissipation in porous membranes resulting from the presence of air and find it to be significant and tunable depending on the sample surface-to-volume ratio. These results provide a new insight into the heat transport at the nanoscale, with potential implications for future design of silicon-based devices for, e.g., energy harvesting, effective heat dissipation in sensors, optomechanics, photonics, requiring operation at high temperatures.

## Methods

### Two-laser Raman thermometry

**Fig. 6:**
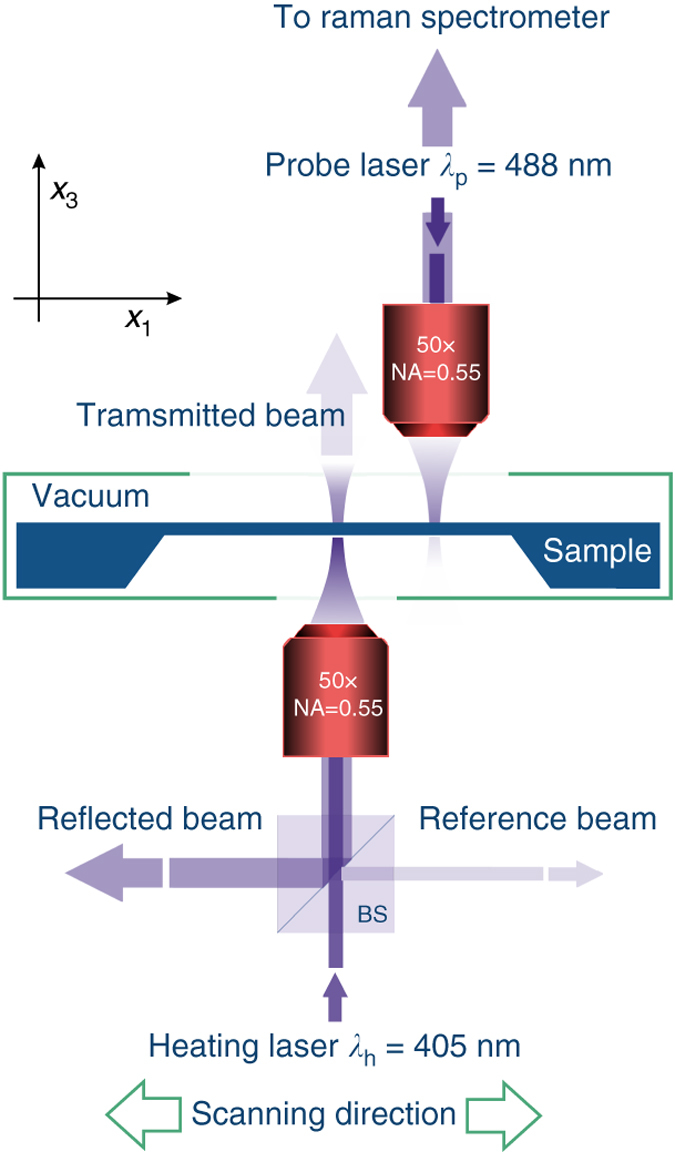
Schematics of the two-laser Raman thermometry experiment. The setup is based on the triple-grating Raman spectrometer (T64000, Horiba) and the vacuum temperature controlled microscope stage (THMS350V, Linkam). The non-polarising cube BS and three powermeters are used to determine the absorbed power *P*_0_ from intensities of the incident, transmitted and reflected laser beams

**Fig. 7:**
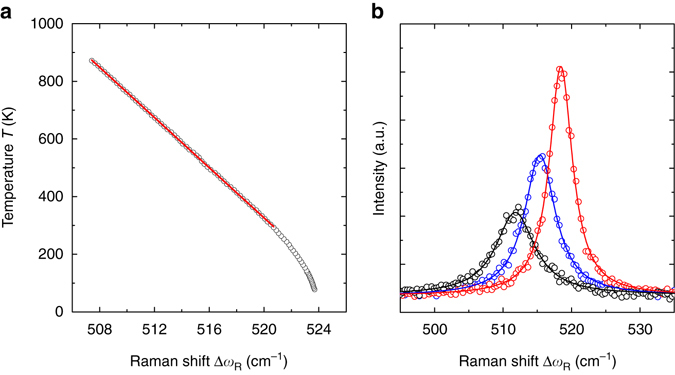
The two-laser Raman thermometry calibration. **a** Temperature as a function of silicon longitudinal optical phonon frequency. The *circles* indicate experimental data points. The *solid line* stands for the linear fit with slope of -43.43 ± 0.05 K(cm^−1^)^−1^ calculated for data ranging between 300 and 870 K. **b** Representative Raman spectra obtained in vacuum for S1 at different distances *r* from the heating spot; *black*, *blue* and *red circles* correspond to *r* = 10, 20 and 40 μm, respectively. *Solid lines* indicate Lorentzian function fits of the corresponding experimental data

Figure 6 shows schematically the concept of the 2LRT experiment performed on porous membranes. Light emitted by the fibre-coupled continuous wave laser operating at *λ*_h_ = 405 nm is focused onto the sample from the bottom by a long working distance microscope objective (×50 and NA = 0.55) acting as a Gaussian heat source with a waist size of about 2 μm. The absorbed power *P*_0_ is measured on site for each sample as the difference between incident and transmitted plus reflected light intensities probed by a calibrated system based on a cube non-polarising beam splitter (BS) with an error of Δ*P*_0_ = 2%. The second laser beam with *λ*_p_ = 488 nm is focused on the sample from the top by the microscope objective (×50 and NA = 0.55) and works as a temperature probe. The sample is mounted in a vacuum chamber, which is fixed to the heating beam unit and motorised scanning stage. The latter is fixed in *x*_3_ (vertical) direction and allows scanning in the *x*_1_ *x*_2_ (Fig. 1b) plane with a spatial resolution of 0.2 μm. The latter value, together with the probe beam spot diameter defined as 1.22*λ*_p_/NA ≈ 1.08 μm, results in the total spatial uncertainty of the experiment Δ*r* ≈ 0.55 μm. The spectral position of the longitudinal optical phonon (LO) of silicon (Δ*ω*_R_(300 K) = 520.7 cm^−1^) is used as a temperature reference. Figure 7a displays a 2LRT temperature calibration curve from which the temperature coefficient between 300 and 870 K was determined as d*T*/dΔ*ω*_R_ = -43.43 ± 0.05 K (cm^−1^)^−1^. Figure 7b shows Raman spectra and their Lorentzian fits of the S1 sample at different relative positions *r* between the heating and probe lasers. The total temperature uncertainty was calculated using the error of the temperature coefficient and standard error of the Lorentzian function fit performed for each point. The uncertainties in the measurements were assessed through error propagation of the equation determining the thermal conductivity of the membranes *κ* = *αT* ^−*β*^, where *α*, *β* and their standard errors Δ*α* and Δ*β*, respectively, are determined by fitting experimental data by means of Eq. (2). The total error of the thermal conductivity was determined from a formula Δ*κ* = ((∂*κ*/∂*α*)^2^Δ*α* ^2^ + (∂*κ*/∂*β*)^2^Δ*β* ^2^)^1/2^. To minimise the influence of the probe laser beam on the measurement, its power is set below the value (typically <2% of *P*_0_) that results in a measurable temperature rise. In principle, for membranes placed on a thick non-circular frame the assumption of the 2D map radial symmetry is valid when the line scan is substantially shorter than the membrane lateral size.

### Thermally generated charge carriers

2LRT enables measurements of *κ* at high temperatures where thermally excited free charge carriers may contribute to the total measured thermal conductivity in two counteracting effects^27^. The first decreases the lattice thermal conductivity due to electron–/hole–phonon scattering, while the second increases the total thermal conductivity due to the presence of additional heat carriers. Both effects strongly depend on the carrier concentration. In our case, according to bulk silicon data^48^ the expected carrier concentration at about 900 K would be below 10^18^ cm^−3^. This value, according to ref. ^27^ renders the electron–/hole–phonon scattering contribution negligible. At the same time the additive effect of the electronic contribution is expected to be small^26^. If the electronic contribution has no size/pores-induced reduction it would account for about 0.3 W m^−1^ K^−1^ of the total thermal conductivity at 900 K, which is within the experimental error for the data of all samples. This is the upper expected value which, due to the high surface-to-volume ratio, can be further decreased. In principle, for bulk silicon the electronic contribution to the thermal conductivity is considered significant at temperatures above 1000 K. In our case this effect would be manifested in the 2LRT logarithmic temperature profile as a mismatch between data points and the dependence given by Eq. (2) gradually increasing with temperature.

### Photo-generated charge carriers

The electron–hole lifetime *τ*_b_ in bulk silicon, depending on doping, purity and temperature can take values from micro to milliseconds^42, 49, 50^. Nevertheless, the effective charge carrier lifetime 1/*τ*_eff_ = 1/*τ*_b_ + 1/*τ*_s_ can be much shorter due to the surface recombination characterised by the surface lifetime *τ*_s_. For the 250 nm thick membrane, carrier diffusivity *D* = 25 cm^2^ s^−1^ and surface recombination velocity *S* = 8700 cm s^−1^ we estimate *τ*_eff_ ≈ *τ*_s_ = 1.4 ns^49, 50^. Thus, the diffusion length takes value of *L* = $\sqrt {D_{T_{\rm eff}}}$ = 1.9 μm. Therefore, and due to the size of the heating spot, we determine *κ* from the experimental data obtained for *r* > 3 μm. In the case of the probe spot, the absorbed laser power is typically lower than *P*_p_ = 50 mW then, considering a 100% efficiency, we estimate a generation rate of *G* = *P*_p_/*E*_ph_ ≈ 1.2×10^14^ s^−1^, where *E*_ph_ ≈ 4.1×10^−19^ J is the photon energy at *λ*_p_ = 488 nm. Taking the probe spot diameter as 1 μm, membrane thickness 250 nm and carrier lifetime as 1.4 ns we estimate the excess carrier concentration to be about 8.8×10^17^ cm^−3^. This value goes below the critical value of 10^19^ cm^−3^, above which electron–/hole–phonon scattering contribution has to be considered^27^. We note that both *τ*_eff_ and *L* can be further decreased by the presence of holes in porous membranes.

### Sample preparation

The positive electron beam resist AR-P 6200 (Allresist) was spun at 4000 r.p.m. for 1 min, followed by 1 min bake at 150 °C on a hot plate. Electron beam lithography (Raith 150-TWO) was performed at 30 kV acceleration voltage. After development in AR 600-546 (Allresist) for 1 min, the samples were post-baked for 1 min at 130 °C on a hot plate. This step additionally hardened the mask before the reactive ion etching. The pattern was transferred to silicon using the Bosch process (Alcatel AMS-110DE). The source power was set to 500 W and the flow of SF_6_ and C_4_F_8_ gases was of 150 and 100 sccm, respectively, and the etching time was of 60 s. Finally, after pattern transfer the samples were placed in a plasma system (PVA Tepla), and cleaned in 50 sccm O_2_ at 400 W for 1 min. The rms surface roughness of the holes was measured by high-resolution SEM (Magellan 400L) to be about 2 nm. The diameter variation of the holes was determined from SEM images to be Δ*d* = ±5 nm. Further details regarding the fabrication process are reported in ref. ^51^.

### Data availability

The data that support the findings of this study are available from the corresponding authors upon request.
